# Polarization Attack on Continuous-Variable Quantum Key Distribution with a Local Local Oscillator

**DOI:** 10.3390/e24070992

**Published:** 2022-07-18

**Authors:** Yun Shao, Yan Pan, Heng Wang, Yaodi Pi, Yang Li, Li Ma, Yichen Zhang, Wei Huang, Bingjie Xu

**Affiliations:** 1 Science and Technology on Communication Security Laboratory, Institute of Southwestern Communication, Chengdu 610041, China; shaoyun@pku.edu.cn (Y.S.); py_swjtu@foxmail.com (Y.P.); wanghg1991@163.com (H.W.); jeremypi@163.com (Y.P.); yishuihanly@pku.edu.cn (Y.L.); mali0878@163.com (L.M.); 2 State Key Laboratory of Information Photonics and Optical Communications, Beijing University of Posts and Telecommunications, Beijing 100876, China; zhangyc@bupt.edu.cn

**Keywords:** continuous variable, quantum key distribution, local local oscillator, phase reference, polarization attack

## Abstract

The estimation of phase noise of continuous-variable quantum key distribution protocol with a local local oscillator (LLO CVQKD), as a major process in quantifying the secret key rate, is closely relevant to the intensity of the phase reference. However, the transmission of the phase reference through the insecure quantum channel is prone to be exploited by the eavesdropper (Eve) to mount attacks. Here, we introduce a polarization attack scheme against the phase reference. Presently, in a practical LLO CVQKD system, only part of the phase reference pulses are measured to compensate for the polarization drift of the quantum signal pulses in a compensation cycle due to the limited polarization measurement rate, while the other part of the phase reference pulses are not measured. We show that Eve can control the phase noise by manipulating the polarization direction of the unmeasured phase reference to hide her attack on the quantum signal. Simulations show that Eve can obtain partial or total key rates information shared between Alice and Bob as the transmission distance increases. Improving the polarization measurement rate to 100% or monitoring the phase reference intensity in real-time is of great importance to protect the LLO CVQKD from polarization attack.

## 1. Introduction

In recent years, theoretical and experimental investigations of quantum key distribution for continuous variable (CVQKD) have increased tremendously [1,2]. CVQKD allows two legitimate communication parties, conventionally referred to as Alice and Bob, to share a common secret key encoded in continuous variables, for which the information-theoretical security is guaranteed by the laws of quantum mechanics. In particular, the Gaussian-modulated coherent-state (GMCS) protocol [3,4], as the most widely implemented CVQKD protocol, has the advantage of compatibility with classical coherent communication infrastructures. This protocol has demonstrated the secret key transmission up to over a 200-km optical fiber [5], and has achieved a field test over a 50-km commercial fiber [6]. At present, the GMCS CVQKD protocol is proved to be secure against the collective attacks and coherent attacks [7,8,9,10,11,12]. Moreover, the composable security proofs of the protocol have been proposed and improved [13,14,15,16]. However, theoretical description used for security proofs may not necessarily faithfully describe the actual setup. Therefore, bridging the gap between theoretical model and practical system is still required to build a robust implementation of quantum cryptography in practical use.

In fact, the practical security problem is a central challenge in all kinds of QKD protocols. Due to the technological imperfection in a QKD system, potential exploitable loopholes are opened for the eavesdropper Eve to launch attacks. In a practical transmission local oscillator (LO) CVQKD system, the LO is generated from Alice and transmitted to Bob through the insecure quantum channel for ease of coherent detection of the quantum signal. In this case, Eve can manipulate the LO to compromise the security of the system severely [17,18,19,20,21]. In order to avoid Eve’s access to the LO, an intriguing local LO protocol for CVQKD (LLO CVQKD) has been proposed and demonstrated [22,23,24], in which the LO is generated on Bob’s side. To date, considerable research have been conducted to improve the protocol [1,25], and a high-rate LLO CVQKD based on Gaussian modulation up to 7.04 Mbits/s over 25-km optical fiber in the asymptotic limit [26]. More recently, the key rate based on discrete modulation CVQKD (in comparison to Gaussian modulation) has been improved by an order of magnitude [27]. Despite the outstanding superiority of the LLO CVQKD in simplifying the hardware required and circumventing the LO attacks, its performance improvement is still severely retarded by the relatively high phase noise [28,29]. As a realistic option, one can use the trusted phase noise model to significantly improve the phase noise tolerance of the LLO CVQKD, in which part of the phase noise that can be locally calibrated by Bob is moved from the channel-added noise to the detector-added noise to get a better QKD performance [30]. For example, with some typical parameters, the transmission distance of the LLO CVQKD based on Gaussian modulation is limited to 40-km. Then, using the trusted phase noise model one can increase the corresponding maximum transmission distance by more than 65% and the secret key rate at the transmission distance of 25-km by more than 60% with the same simulation parameters [30]. Nevertheless, in a practical LLO CVQKD system, a relatively weak classical phase reference is generated from the signal laser and propagates along with the quantum signal from Alice to Bob to establish a reliable phase relationship between the quantum signal and the LO. This configuration will inevitably leave a security loophole for Eve to attack the phase reference [29,31]. Therefore, it is an ongoing task to search the security vulnerabilities and propose appropriate countermeasures.

Here, we introduce a polarization attack scheme against the LLO CVQKD protocol, inspired by the polarization attack on the transmission LO CVQKD [32]. This attack arises from the limited compensation rate in the polarization compensation process for the quantum signal. In a practical LLO CVQKD system, the phase reference is used to compensate for the polarization drift between the quantum signal and the LO. It is shown that Eve can use the system imperfection to hide her attack on the quantum signal by manipulating the polarization of the phase reference. The security of the LLO CVQKD system can be fully compromised without corresponding countermeasures.

This paper is organized as follows: in Section 2, we review the LLO CVQKD scheme and the trusted phase noise model, where the calculation formulas for secret key rate are presented. In Section 3, we discuss the polarization attack scheme against the phase reference as well as the countermeasures. Finally, the conclusion is given in Section 4.

## 2. Trusted Phase Noise Model for LLO CVQKD

In the following, we first review the trusted phase noise model for LLO CVQKD protocol, which will be essential for the analysis in the next section. We then present the calculations of the asymptotic secret key rate for CVQKD under the collective attack.

**Figure 1:**
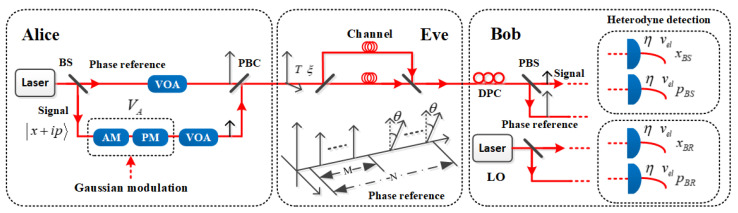
Schematic representation of the time-polarization multiplexing LLO CVQKD scheme. BS is the optical beam splitter, AM is the optical amplitude modulator, PM is the optical phase modulator, VOA is the variable optical attenuator, PBC is the optical polarization beam combiner, DPC is the dynamic polarization controller, and PBS is the optical polarization beam splitter.

For simplicity, we assume the time-polarization multiplexing system for GMCS LLO CVQKD protocol based on heterodyne detection [33,34] are adopted, as illustrated in Figure 1. Alice prepares few-photon coherent state |x+ip〉 as quantum signal, in which the two orthogonal quadratures x and p are continuously modulated with Gaussian distribution centered on zero and with variance $V_{A}N_{0}$. Here, $N_{0}$ is the shot noise variance, and all noise variances in this paper are expressed in shot noise units (SNU). The coherent-state quantum signal is interleaved with the time-delayed phase reference and transmitted through an untrusted quantum channel that is characterized by transmittance T and excess noise ξ. On the receiver side, Bob performs heterodyne detection using a locally generated LO pulses to measure both quadratures of the quantum signal simultaneously. He also performs heterodyne detection to measure both quadratures of the phase reference simultaneously so as to estimate the phase rotation of the quantum signal between Alice’s and Bob’s independent lasers frames. That is reasonable because the phase reference and the quantum signal are generated from the same laser and experience similar environmental effects. The coherent detector features an efficiency η and electronic noise $\nu_{el}$. After Alice and Bob obtain the correlated Gaussian variables as raw key, they can perform postprocessing, including parameter estimation, error correction, and privacy amplification, to get a secret key.

Based on the scheme described above, the phase noise for the quantum signal can be estimated by [21,30,35]

(1) $$\xi_{\mathrm{phase}}\approx \xi_{\mathrm{error}}=V_{A}\left(\frac{\chi +1}{E_{R}^{2}}\right).$$

where the phase noise $\xi_{\mathrm{phase}}$ is dominated by the phase reference measurement noise $\xi_{\mathrm{error}}$. Here, $E_{R}$ is the amplitude of the phase reference on Bob’s side, χ is the total added noise imposed on the phase reference given by [30,31,35]

(2) $$\chi =\frac{1-T}{T}+\varepsilon_{0}+\frac{2-\eta +2\nu_{el}}{T\eta }$$

where $\varepsilon_{0}$ is the excess noise of the phase reference with typical value $\varepsilon_{0}=0.002$ [36]. In the trusted phase noise model [30], part of the phase reference measurement noise associated with the detector efficiency η and the electronic noise $\nu_{el}$ of Bob’s detector as well as the phase reference intensity on the receiver side that can be locally calibrated by Bob is considered to be trusted in order to get a higher secret key rate and longer transmission distance. Therefore, Equation (1) can be decomposed as:

(3) $$\xi_{\mathrm{phase}}=\xi_{\mathrm{phase}}^{U}+\frac{\xi_{\mathrm{phase}}^{T}}{T},\;$$

According to Equations (1)–(3), we have

(4) $$\xi_{\mathrm{phase}}^{U}=\frac{V_{A}\left(1+T\varepsilon_{0}\right)}{TE_{R}^{2}},$$

(5) $$\xi_{\mathrm{phase}}^{T}=\frac{V_{A}\left(2-\eta +2\nu_{el}\right)}{\eta E_{R}^{2}}$$

In this regard, the added noises for the quantum signal can be modeled as follows [30]:

(6) $$\chi_{\mathrm{line}}=\frac{1}{T}-1+\xi_{\mathrm{tot}}-\frac{\xi_{\mathrm{phase}}^{T}}{T},$$

(7) $$\chi_{\mathrm{het}}=\frac{2-\eta +2\nu_{el}}{\eta }+\xi_{\mathrm{phase}}^{T},$$

(8) $$\chi_{\mathrm{tot}}=\chi_{\mathrm{line}}+\frac{\chi_{\mathrm{het}}}{T}.$$

In Equation (6), $\chi_{\mathrm{line}}$ represents the total channel added noise referred to the channel input, in which $\xi_{\mathrm{tot}}$ stands for the total excess noise obtained from the parameters estimation procedure, and mainly consists of the following parts [30]:

(9) $$\xi_{\mathrm{tot}}=\xi_{0}+\xi_{\mathrm{AM}}+\xi_{\mathrm{LE}}+\xi_{\mathrm{ADC}}+\xi_{\mathrm{phase}}^{U}+\frac{\xi_{\mathrm{phase}}^{T}}{T}.$$

Here, $\xi_{0}$ is the system excess noise stemming from the unidentified or unprotected sources [28]. $\xi_{\mathrm{AM}}$ is the modulation noise that can be expressed as [35] $\xi_{AM}={\left(E_{S\max}^{A}\right)}^{2}10^{-d_{dB}/10}$, where $E_{S\max}^{A}\approx \sqrt{10V_{A}}$ quantifies the maximal amplitude of the quantum signal. $\xi_{\mathrm{ADC}}$ is the analog-to-digital quantization noise which satisfies [34] $\xi_{\mathrm{ADC}}\geq {\left(E_{S\max}^{A}\right)}^{2}/\left(12\times 2^{n}\right)$, where $E_{S\max}^{A}$ stands for the maximal amplitude of the quantum signal to be modulated. $\xi_{\mathrm{phase}}^{U}$ is the untrusted part of the phase noise referred to the channel input, and $\xi_{\mathrm{phase}}^{T}$ corresponds to the trusted part of the phase noise referred to Bob’s input. In Equation (7), $\chi_{\mathrm{het}}$ represents the detection added noise referred to Bob’s input. Equation (8) represents the total added noise referred to the channel input.

**Figure 2:**
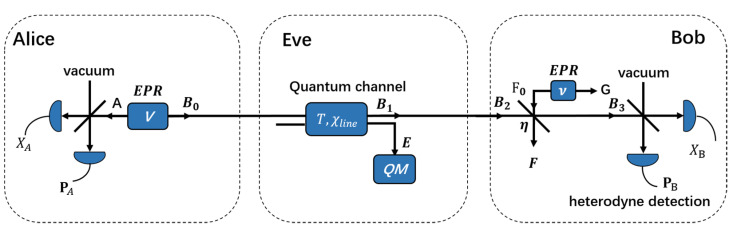
Schematic representation of the entanglement-based description of the CVQKD protocol. Alice’s Gaussian modulation of the coherent state is modelled by a heterodyne detection of one half of an EPR state with variance *V*. Bob’s detector noise is modeled by a beam splitter with transmission η, and the electronic *v_el_* is modeled by a EPR state with variance *v*. The QM stands for Eve’s quantum memory.

It is known that the above prepare-and-measure CVQKD scheme is equivalent to the entanglement-based protocol, as outlined in Figure 2, for which the security against collective attacks has been strictly proved [37]. The asymptotic secret key rate of the LLO CVQKD in the context of reverse reconciliation can be expressed as [37]

(10) $$K=\beta I_{\mathrm{AB}}-\chi_{\mathrm{BE}},$$

where β is the reconciliation efficiency, $I_{\mathrm{AB}}$ is Shannon mutual information between Alice and Bob, and $\chi_{\mathrm{BE}}$ is the Holevo information bound between Eve and Bob. The mutual information can be given by [37,38]

(11) $$I_{\mathrm{AB}}=\log_{2}\frac{V+\chi_{\mathrm{tot}}}{1+\chi_{\mathrm{tot}}},$$

(12) $$\chi_{\mathrm{BE}}=\overset{2}{\underset{i=1}{\sum }}G\left(\frac{\lambda_{i}-1}{2}\right)-\overset{5}{\underset{i=3}{\sum }}G\left(\frac{\lambda_{i}-1}{2}\right),$$

with $V=V_{A}+1$ is the variance of the thermal state that Alice sent to Bob, and $G\left(x\right)=\left(x+1\right)\log_{2}\left(x+1\right)-\log_{2}x$. The symplectic eigenvalues can be expressed as

$$\lambda_{1,2}^{2}=\frac{1}{2}\left[A\pm \sqrt{A^{2}-4B}\right],$$

$$\lambda_{3,4}^{2}=\frac{1}{2}\left[C\pm \sqrt{C^{2}-4D}\right],$$

(13) $$\lambda_{5}=1.$$

where

$$A=V^{2}\left(1-2T\right)+2T+T^{2}{\left(V+\chi_{\mathrm{line}}\right)}^{2},$$

$$B=T^{2}{\left(V\chi_{\mathrm{line}}+1\right)}^{2},$$

$$C=\frac{1}{{\left[T\left(V+\chi_{\mathrm{tot}}\right)\right]}^{2}}\left[A\chi_{\mathrm{het}}^{2}+B+1+2\chi_{\mathrm{het}}\times \left(V\sqrt{B}+T\left(V+\chi_{\mathrm{line}}\right)\right)+2T\left(V^{2}-1\right)\right],$$

(14) $$D={\left(\frac{V+\sqrt{B}\chi_{\mathrm{het}}}{T\left(V+\chi_{\mathrm{tot}}\right)}\right)}^{2}.$$

## 3. Polarization Attack on the Phase Reference

In this section, we aim to discuss the security vulnerability and corresponding potential hack attack caused by technological imperfection of a practical LLO CVQKD system such as polarization turbulence of the quantum signal. Generally, the phase reference in LLO CVQKD system is required to transmit through the quantum channel to monitor and compensate for the phase and polarization drift of the quantum signal, which, however, could be used by Eve to mount attacks, such as the polarization attack.

In the previous study for the transmission LO CVQKD system, as the SNU plays an important role in CVQKD [39], a quantum hacking method was identified where Eve can attack the unmeasured LO pulses to control and tamper the practical SNU by using the limited compensation rate during the polarization compensation for the signal pulses [32]. Unlike the transmission LO CVQKD protocol, in the LLO CVQKD protocol, since the LO pulses are generated by Bob on the receiver side, potential attacks against the LO pulses will be ruled out. For a practical LLO CVQKD system, in order to establish a stable coherent detection for the quantum signal, aligned laser polarization directions between the quantum signal and the LO pulse are desired. However, the polarization drift of the quantum signal will reduce the efficiency of coherent detection owing to random perturbation in the quantum channel. Therefore, a polarization-drift compensation process is particularly necessary. From a practical point of view, in the LLO CVQKD system, since the quantum signal is too weak to identify its polarization direction on the receiver side, the weak classical phase reference is used to perform polarization measurement and compensation for the quantum signal. Ideally, a real-time polarization measurement and feedback control of each pulse for the phase reference and quantum signal would compensate for the polarization drift. More specially, as the polarization measurement rate of current commercial devices is much lower than the repetition frequency of the LLO CVQKD system [32], the polarization compensation in a practical system is performed by measuring part of the phase reference pulses in a compensation cycle. It is assumed that the polarization of the measured pulses is the same as that of the unmeasured pulses in a compensation cycle. Nevertheless, this approach will bring security risk, because Eve can manipulate the polarization direction of the unmeasured phase reference pulses, which would result in the discrepancy of polarization between the unmeasured pulses and the measured pulses. In the following discussion, we will show that in the context of LLO CVQKD system, Eve has the ability to attack the unmeasured phase reference pulses to change the trusted part of the phase noise by manipulating the laser polarization of the unmeasured phase reference pulses in the quantum channel, which will make Alice and Bob overestimate the secret key rate.

As shown in Figure 1, in a time-polarization multiplexing LLO CVQKD system, on Alice’s side, the parallel polarized signal pulse and phase reference pulse are recombined by a polarization beam combiner (PBC) to orthogonal polarization modes. After propagation through the lossy channel, a polarization-drift compensation process for the signal pulse and the phase reference pulse is implemented on the receiver side. First, in a polarization compensation cycle with *N* phase reference pulses, Bob selects *M* pulses to measure their polarization to determine the polarization drift from the target polarization. Second, a feedback signal based on the above measured results is generated to modulate the polarization controller to compensate for the polarization drift of the signal pulse and the phase reference pulse. Then the signal pulse and the phase reference pulse are demultiplexed and split into two paths by the polarization beam splitter (PBS), and made to interfere with the LO pulse separately on a balanced heterodyne detector.

In the trusted phase noise model for LLO CVQKD protocol, it is usually assumed that the phase reference intensity $E_{R}^{2}$ is measured and the trusted part of the phase noise $\xi_{\mathrm{phase}}^{T}$ is calibrated before the QKD run. In this case, Bob has no idea about $\xi_{\mathrm{phase}}^{T}$ when the intensity of the phase reference fluctuates during the QKD run. Consequently, Alice and Bob will get a false key rate if Eve can manipulate the intensity of the phase reference by changing its polarization direction during the key distribution process, while Bob still adopts the previously measured intensity to estimate the trusted part of the phase noise, as illustrated in Figure 1. To perform this attack, during the trusted phase noise calibration stage, Eve intercepts all the quantum signal and the phase reference sent by Alice at the channel input, and then separates them into her own two perfect quantum channel. For the phase reference pulse within one compensation cycle, Eve makes the polarization direction of the N−M pulses deviate from the polarization direction of the M pulses whose polarization are measured for polarization drift compensation. Here, we use θ to represent the misalignment angle between them. After the polarization compensation, the intensity projection of the N−M pulses in the main axis of the PBS at Bob’s side thus becomes $E_{R}^{2}\cos^{2}\theta$. Following the scheme described above in Figure 1, according to Equation (5), the trusted part of the phase noise under the attack can be expressed as

(15) $$\xi_{\mathrm{phase}}^{T-\mathrm{attack}}=V_{A}\left[\frac{2-\eta +2\nu_{el}}{\eta [kE_{R}^{2}+\left(1-k\right)E_{R}^{2}\cos^{2}\theta ]}\right]=\xi_{\mathrm{phase}}^{T}\left[\frac{1}{k+\left(1-k\right){cos}^{2}\theta }\right].$$

where k=M/N is the ratio of the measured pulses to the compensation cycle pulses, which is named as polarization measurement rate (PMR).

Next, when Alice and Bob start the key distribution process, Eve can reduce θ to narrow the deviation of the polarization directions between the measured pulses (M) and the unmeasured pulses (N−M). This meant that the actual average intensity of the phase reference projected on the main axis of the PBS will be higher than its initial calibrated value. Note that the maximum change of the average intensity corresponds to the reduction of θ value to zero. In this case, one can apply Equation (15) to obtain the reduction of the trusted part of the phase noise, which can be written as

(16) $$\Delta \xi_{\mathrm{phase}}^{T}=\xi_{\mathrm{phase}}^{T-\mathrm{attack}}{|}_{\theta =\theta }-\xi_{\mathrm{phase}}^{T-\mathrm{attack}}{|}_{\theta =0}=\xi_{\mathrm{phase}}^{T}\left[\frac{1}{k+\left(1-k\right){cos}^{2}\theta }\right]-\xi_{\mathrm{phase}}^{T}.$$

One can find that the larger the misalignment angle θ controlled by Eve, the more the reduction of the trusted phase noise. It is worth noting that simply reducing the trusted phase noise does not necessarily imply that it will help Eve steal information about the quantum signal. This is because the total excess noise $\xi_{tot}$ is estimated from the parameters estimation procedure. The reduction of the trusted phase noise will lead to the reduction of the total excess noise, and the key rate information available to Eve can be estimated and discarded by Alice and Bob through the privacy amplification process [4]. Therefore, for purposes of getting the encoded information, Eve has to increase her attack on the quantum signal during the key distribution process, which will inevitably introduce excess noise $\xi_{\mathrm{attack}}$. In this case, Eve can use the reduced part of the trusted phase noise to compensate for the introduced excess noise to hide her attack on the quantum signal, and gain information when the total excess noise is within the maximum tolerable excess noise. For simplicity of analysis, we assume that the reduction of the trusted phase noise is equal to excess noise introduced by Eve, i.e.,

(17) $$\xi_{\mathrm{attack}}=\Delta \xi_{\mathrm{phase}}^{T}.$$

Hence, when the phase reference is attacked, the added excess noise for the LLO CVQKD system under the trusted phase noise model can be expressed as

(18) $$\chi_{\mathrm{line}}^{\mathrm{attack}}=\frac{1}{T}-1+\xi_{\mathrm{tot}}-\frac{\xi_{\mathrm{phase}}^{T}}{T}+\frac{\xi_{\mathrm{attack}}}{T},$$

(19) $$\chi_{\mathrm{het}}^{\mathrm{attack}}=\frac{2-\eta +2\nu_{el}}{\eta }+\xi_{\mathrm{phase}}^{T}-\Delta \xi_{\mathrm{phase}}^{T},$$

(20) $$\chi_{\mathrm{tot}}^{\mathrm{attack}}=\chi_{\mathrm{line}}^{\mathrm{attack}}+\frac{\chi_{\mathrm{het}}^{\mathrm{attack}}}{T}.$$

Therefore, under the polarization attack, the total channel-added noise is increased while the detector-added noise is reduced, which will cause Alice and Bob to overestimate the secret key rate. Combining the above scheme with the calculations from Equations (10)–(14), we can get the key rate under the polarization attack.

**Figure 3:**
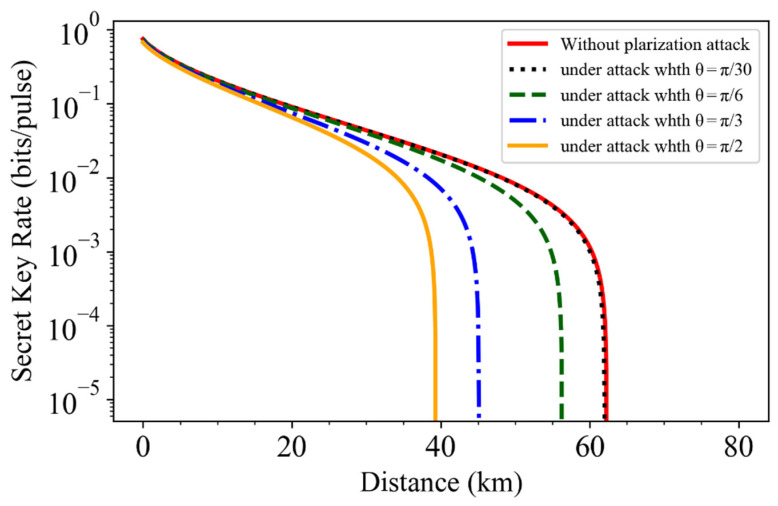
Simulations of secret key rate for the LLO CVQKD system under the trusted phase noise model. The right solid red line represents the result without attack. The black dotted line, green dashed line, blue dashed-dotted line, and left orange solid line represent the results under polarization attack when the misalignment angle θ is π/20, π/6, π/3, and π/2, respectively, where the PRM is fixed with k=0.5.

In Figure 3, we simulate the secret key rate results for the LLO CVQKD system under the trusted phase noise model with a fixed PMR k=0.5. The other typical parameters, as used in Refs. [28,29,30,31,34], are as follows: reconciliation efficiency β=0.95, detector efficiency η=0.5, modulation variance $V_{A}=4$, electronic noise $\nu_{el}=0.1$, attenuation coefficient α=0.2 dB/km, phase reference intensity $E_{R}^{2}=1000$, system excess noise $\xi_{0}=0.01$, ADC quantization number n=10, AM dynamics $d_{dB}=40$, and finite extinction ratios $R_{e}=40\;\mathrm{dB}$ and $R_{P}=30\;\mathrm{dB}$. The right solid red line represents the result without polarization attack, where the maximum transmission distance is larger than 60 km. Compared to the result without attack, the phase reference polarization attack can fully constitute threat to the security of the LLO CVQKD protocols. One can find that Eve’s intercepted information of the quantum signal is proportional to the misalignment angle θ of the unmeasured phase reference pulse. The left orange solid line represents the extreme polarization attack case where the misalignment angle is θ=π/2. In this case, the real maximum transmission distance is dropped to less than 40 km, that is Eve can obtain partial or total key rate when the transmission distance is lower or higher than 40 km. We also calculate the secret key rate under different situations at the transmission distance of 30 km. It is shown that Eve can steal 8%, 32%, and 52% of the key information shared by Alice and Bob when the misalignment angles are θ=π/6, θ=π/3, and θ=π/2, respectively. Moreover, the black dotted line shows that for small misalignment angle (θ=π/30) the simulation approaches that of the case without polarization attack.

**Figure 4:**
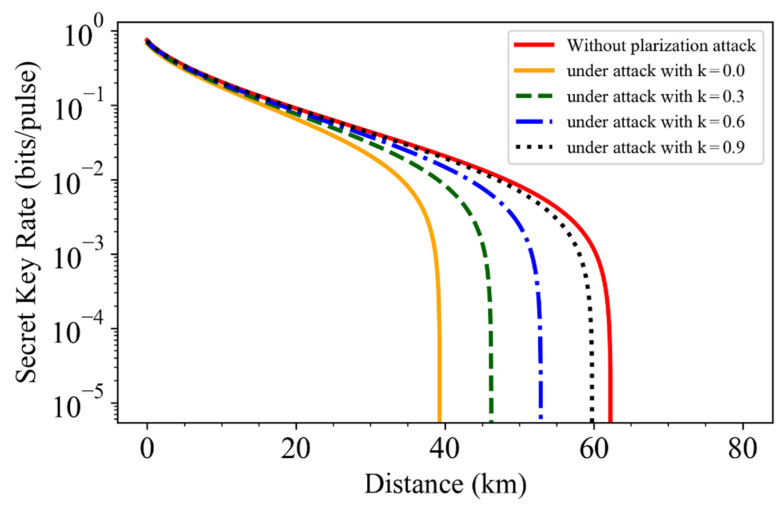
Simulations of secret key rate for the LLO CVQKD system under the trusted phase noise model. The right red solid line represents the result without attack. The black dotted line, blue dashed-dotted line, green dashed line and right orange solid line represent the results under polarization attack when the PMR *k* is 0, 0.3, 0.6 and 0.9, respectively, where the misalignment angle is fixed with θ=π/4.

We further simulate the secret key rate at different PMR for a fixed misalignment angle θ=π/4. The other simulation parameters are the same as that in Figure 3. One can see from Figure 4 that the larger the PMR, the more information about the quantum signal Eve stole. The left orange solid line represents the results with PMR=0, where all the phase reference pulses can be manipulated by Eve to change the polarization to reduce the trusted phase noise. It can be speculated that the polarization attack can be prevented as the PMR increases to 100%. From the simulations one can find that Eve can steal 3%, 10%, 23%, and 52% of the quantum signal held by Alice and Bob when the PMR are k=0.9, k=0.6, k=0.3, and k=0, respectively.

The above described attack scheme uncovers the importance of monitoring the intensity of the phase reference in real-time, which has been discussed in previous studies [31]. Mover, based on the analysis in Figure 4, one can find that improving the PMR of the phase reference pulse to 100% is also an effective way to protect the LLO CVQKD protocol from the polarization attack.

Next, let us look into the difference between the phase reference intensity attack [31] and the proposed phase reference polarization attack. Indeed, in the above two attack strategies, Eve essentially steals the quantum signal by manipulating the intensity of the phase reference. However, there are differences in both the attack schemes and the countermeasures. First, in the former attack scheme, Eve increases the intensity of the whole phase reference pulses directly using an intensity amplifier, while in the latter attack scheme, Eve does this by attacking the polarization compensation module to manipulate the polarization of the unmeasured phase reference pulses in a compensation cycle. Second, the countermeasures against the attacks are not exactly the same. For the latter attack scheme, in addition to monitoring the intensity of the phase reference in real time, one could improve the PMR to resist the attack.

## 4. Conclusions

In summary, we have studied the practical security of LLO CVQKD system related to phase reference. In a practical system, part of the phase reference pulses are used to measure and compensate for the polarization drift of the signal pulses. We have shown that the limited PMR for the phase reference will leave a security loophole, which can be exploited by Eve to mount attacks. We have proposed a polarization attack scheme, from which Eve can reduce the trusted phase noise to compensate for the introduced attack noise by manipulating the polarization of the unmeasured phase reference pulses. The simulations show that the lager the misalignment angle controlled by Eve and the smaller the PMR, the more information Eve can steal. To improve the practical security of the system, on the one hand, one can increase the PRM to 100%; on the other hand, one can monitor the intensity of the phase reference in real time.
